# Ketogenic Diet as a Treatment for Super-Refractory Status Epilepticus in Febrile Infection-Related Epilepsy Syndrome

**DOI:** 10.3389/fneur.2019.00423

**Published:** 2019-04-26

**Authors:** Pan Peng, Jing Peng, Fei Yin, Xiaolu Deng, Chen Chen, Fang He, Xiaole Wang, Shiqi Guang, Leilei Mao

**Affiliations:** ^1^Department of Pediatrics, Xiangya Hospital of Central South University, Changsha, China; ^2^Hunan Intellectual and Developmental Disabilities Research Center, Xiangya Hospital of Central South University, Changsha, China

**Keywords:** ketogenic diet, febrile infection-related epilepsy syndrome, super-refractory status epilepticus, PICU, EEG

## Abstract

**Background:** Febrile infection-related epilepsy syndrome (FIRES) is a fatal epileptic encephalopathy associated with super-refractory status epilepticus (SRSE). Several treatment strategies have been proposed for this condition although the clinical outcomes are poor. Huge efforts from neurointensivists have been focused on identifying the characteristics of FIRES and treatment to reduce the mortality associated with this condition. However, the role of ketogenic diet (KD) in FIRES is not fully understood.

**Methods:** We performed a retrospective review of patients who met the diagnostic criteria of FIRES, SRSE, and were treated with KD between 2015 and 2018 at the Department of Pediatrics, Xiangya Hospital of Central South University. The following data were recorded: demographic features, clinical presentation, anticonvulsant treatment, timing and duration of KD and follow-up information. Electroencephalography recordings were reviewed and analyzed.

**Results:** Seven patients with FIRES were put on KD (5 via enteral route, and 2 via intravenous line) for SRSE in the PICU. The median age was 8. Four patients were male and 3 were female. Although patients underwent treatment with a median of 4 antiepileptic drugs and 2 anesthetic agents, the status epilepticus (SE) persisted for 7–31 days before KD initiation. After KD initiation, all patients achieved ketosis and SE disappeared within an average of 5 days (IQR 3.5), although there were minor side effects. In 6 patients, a unique pattern was identified in the EEG recording at the peak period. After initiation of KD, the number of seizures reduced, the duration of seizure shortened, the background recovered and sleep architecture normalized in the EEG recordings. The early initiation of KD (at the onset of SE) in the acute phase of patients decreased the mRS score in the subsequent period (*p* = 0.012, *r* = 0.866).

**Conclusions:** The characteristic EEG pattern in the acute phase promoted timely diagnosis of FIRES. Our data suggest that KD may be a safe and promising therapy for FIRES with SRSE, and that early initiation of KD produces a favorable prognosis. Therefore, KD should be applied earlier in the course of FIRES. Intravenous KD can be an effective alternative route of administration for patients who may not take KD enterally.

## Introduction

Febrile infection-related epilepsy syndrome (FIRES) is a rare epileptic encephalopathy of unknown etiology which occurs in patients without active epilepsy or underlying neurological disorders. It is characterized by new onset of refractory status epilepticus (SE) following a short nonspecific febrile illness (1). It is a biphasic disease, with an acute phase of refractory SE that always transforms into super-refractory SE (SRSE, i.e., SE that continues or recurs 24 h or more after the onset of anesthesia). This condition often requires intensive care and management, and it often progresses to a chronic phase which is characterized by refractory epilepsy without an intervening silent period. In the acute phase, the risk of mortality is high. Moreover, survivors are likely to live with a drug-resistant epilepsy and neuropsychological impairment. Nevertheless, few individuals do not experience neurologic sequelae or mild learning difficulties (2). Multiple therapeutic modalities have been reported for this disease, but most of them do not effectively control the high epileptic activity in such patients (3). Therefore, it is imperative to develop FIRES therapies with high efficacy.

Ketogenic diet (KD) contains high-fat, low-carbohydrate, and moderate protein content. It alters the primary cerebral energy supply from glucose to ketone bodies, mimicking the biochemical changes of starvation (4, 5). Although the exact mechanisms of KD's are not well-defined, it is thought that KD has antiepileptic activity, anti-inflammatory effect and neuroprotective activity (6–9) making it a therapeutic target for FIRES. In recent years, several small case studies have highlighted the value of diet in the treatment of FIRES. Results from such studies indicate that diet is not only suitable for acute patient management, but also for long-term management (10, 11). In April 2018, the International Ketogenic Diet Study Group have reached a consensus that KD is particularly useful for FIRES (12).

Despite fatal presentation, high disability and fatality rate in patients with FIRES, this syndrome is rarely diagnosed. Patient with FIRES are often diagnosed using the exclusion diagnosis method, and this explains why patients with FIRES are often diagnosed at a late stage due to lack of early disease markers. Currently, there is inadequate scientific data on the therapeutic efficacy of early initiation of KD for FIRES. Therefore, we aimed to explore strategies for timely recognition of FIRES through EEG recordings. Moreover, the KD therapy was given to 7 patients with FIRES and SRSE to investigate the efficacy and safety of KD as well as determine the effect of early KD treatment on the prognosis of FIRES patients.

## Materials and Methods

### Patients and Approvals

We carried out a retrospective review of 7 patients diagnosed with FIRES (13, 14) and SRSE, and treated with KD at the Department of Pediatrics of Xiangya Hospital in Central South of China between 2015 and 2018. This study was approved by The Central South University Institutional Review Board.

### Clinical Data

The following patient data was collected: demographics, general situations, history of seizures or SE, Glasgow Coma Scale (GCS), total hospital length of stay (LOS), ICU LOS, and antiepileptic drugs (AEDs) used before KD initiation and at discharge. Anesthetic agents, SE duration prior to KD, KD ratio, type and duration of KD, presence of ketones, and KD side effects were also obtained. KD therapy, seizure burden and modified Rankin Scale score (mRS) score at follow-up were collected in the outpatient neurology clinics. The clinical data were supplemented by a retrospective review of the electronic medical records (EMC).

Considering that SE has a subtle or no clinical manifestations in FIRES, its diagnosis requires EEG monitoring. Here, two approaches were adopted to define SE. Before hospital admission, SE was defined by continuous clinical or electrographic seizures lasting 5 min or more and/or recurrent episodes without recovery in between episodes (15). After admission, SE was defined by either a single seizure lasting ≥30 min or recurrent seizures totaling ≥30 min in any 1-h period (16).

All EEG recordings were digital with time-locked synchronized video. Electrodes were positioned in accordance with the international 10–20 system of electrode placement. The EMG activity and ECG were also recorded. All patients were monitored with EEG for 2–15 h immediately after arrival at our PICU. The frequency of EEG monitoring varies, depending on available institutional resources and perceived clinical condition. All EEG recordings were analyzed individually by a board-certified pediatric neurologist and two Asian Epilepsy Academy (ASEPA)-certified electroencephalographers. The following EEG features were analyzed: seizure burden, interictal and periodic epileptiform discharges, background activity, and sleep architecture. Five segments of EEG recordings were reviewed selectively to confirm the specific EEG pattern of FIRES and the effectiveness of KD. We used the first recording at admission to our PICU as the 1# epoch, the last recording prior to initiation of KD as the 2# epoch. The recording at which SE disappeared after KD treatment was recorded as the 4# epoch, and the last recording before discharge as the 5# epoch. The 3# epoch was selected to divide the period between 2 and 4# approximately into two equal parts. All EEG data was stored in original format and was used for review.

Data were analyzed by reviewing of physician notes, laboratory findings, neuroimaging studies, EEG recordings and other supporting data.

### Diet Initiation

Baseline laboratory examinations including complete blood counts, serum carnitine, chemistries, electrolytes, lipids, fatty acids were performed for all patients prior to KD initiation. These parameters were evaluated by a collaborative team of neurologists, critical care medicine personnel and nutrition experts to ensure there were no contraindications to KD therapy. KD was administered through two routes without a fasting period. All patients received enteral KD (EN KD) except 2 patients with severe gastrointestinal dysfunction who were placed on intravenous KD (IV KD). The commercially available ketogenic formula (Jiantong, Kinton medical food. Ltd, Guangzhou, China) with a fat to protein and carbohydrate (in gram scale) ratio of 4:1 were administered via enteral route and mixed with the current formula at the prescribed ketogenic ratio. IV KD comprised of commercially available fat emulsion with medium-chain triglycerides (Lipovenoes MCT, Sino-Swed Pharmaceutical Corp. Ltd, Wuxi, China), dextrose and amino acid solutions (Vamin, Sino-Swed Pharmaceutical Corp. Ltd, Wuxi, China).

The KD was initiated at a low ratio (about 2–3:1 in EN KD, 1:1 in IV KD) and then gradually advanced to a ratio of 3–4:1. The calorie intake of KD was restricted to 1/3 of the estimated diet energy needs (75% of recommended dietary allowance) initially and increased by 1/3 every 1–3 days (slower in IV KD) to full estimated diet energy. This step-by-step approach was adopted to prevent hypertriglyceridemia or hyperamylasemia induced by high intake or infusion of lipids. All dextrose was removed from fluids and all medications were changed to low-carbohydrate forms. Glucose-free solutions such as anticonvulsants and saline were prepared as required. Electrolytes, minerals, vitamins, and micronutrients were prepared at normal concentrations. The enteral preparation was given 6–8 times per day. IV KD was infused continuously over 20 h and stopped for 4 h. When enteric-related complications such as gastrointestinal bleeding and enteroparalysis were resolved, EN KD was initiated and the calorie intake was increased by 1/10–1/5 of the estimated daily intake, adjusted after each 1–2 days to replace IV KD. Electrolytes, arterial blood gases, serum ketone bodies and glucose were measured once KD was initiated.

### Data Analysis

Statistical analysis was performed using the Statistical Package for Social Science (IBM, SPSS Statistics Version 21). Proportions were calculated for continuous variables whereas medians and interquartile ranges (IQRs) were calculated for categorical variables. Pearson correlation analysis was applied for the duration of SE before KD initiation and mRS score at the latest follow-up (the normality test was completed).

## Results

### Clinical Characteristics

In this series, all 7 patients (4 boys and 3 girls) were transferred from other health facilities and diagnosed with FIRES and SRSE in our PICU. All patients had a normal health and psychomotor development in their history. They did not have familial history of seizure or a known neurologic disease. The ages of patients at disease onset were between 1.5 and 13 years. The demographic data, clinical manifestations and management are described in Table 1. Out of 7 patients, 4 had a GCS score < 8 at admission. The hospital LOS ranged from 35 to 86 days (median 46; IQR 18.5; mean 50.3 ± 18.5) and ICU LOS ranged from 19 to 61 days (median 32; IQR 15; mean 32.6 ± 14.7). Among them, no clear etiology could be determined despite extensive analyses ranging from microbiologic, metabolic and autoimmune causes, and early brain Magnetic Resonance Imaging (MRI).

**Table 1:** Patient characteristics and treatment.

	**Patient 1**	**Patient 2**	**Patient 3**	**Patient 4**	**Patient 5**	**Patient 6**	**Patient 7**
Antecedent febrile infection	FUO	URTI	URTI	GE	URTI	FUO	FUO
Latent period^*^	3	5	4	4	2	5	3
Admit GCS score	4	3	9	E4VTM1	E1VTM1	Confusion	Confusion
Duration of SE before KD, *d*	31	12	11	15	11	29	7
No. of AEDs received before KD	5	4	4	5	3	4	4
AEDs received before KD	PHB, CZP, VPA, TPM, OXC	VPA, LEV, OXC, NZP	PHB, VPA, TPM, LEV	PHB, CZP, VPA, TPM, OXC	VPA, TPM, LEV	VPA, LEV, TPM, OXC	VPA, LEV, TPM, OXC
No. of anesthetic agents before KD	2	2	2	2	2	1	1
Anesthetic agents before KD	Midazolam, Propofol	Midazolam, Propofol	Midazolam, Propofol	Midazolam, Propofol	Midazolam, Propofol	Midazolam	Midazolam
Anesthetics assistants before KD	Suf, Cisatracurium Besilate, Vecuronium	/	/	/	Fentanyl	/	/
Other treatments before KD	MP, IVIG, PE	MP, IVIG	IVIG	MP, IVIG	MP, IVIG, PE	IVIG	MP, IVIG
AMV	Yes	No	No	Yes	Yes	No	No
KD ratio	1.2:1 → 4:1	1.5:1 → 3.2:1	2:1 → 2.5:1	1.5:1 → 3:1	0.7:1 → 4:1	2:1 → 3:1	3:1 → 4:1
Type of KD	Parental	Enteral	Enteral	Enteral	Parental	Enteral	Enteral

*AEDs, antiepileptic drugs; CLB, clobazam; CZP, clonazepam; F, female; FUO, fever of unknown origin; GCS, Glasgow Coma Scale; GE, gastroenteritis; IVIG, intravenous immunoglobulin; LEV, levetiracetam; M, male; MP, methylprednisolone; NZP, nitrazepam; OXC, oxcarbazepine; PE, plasma exchange; PHB, phenobarbital; Suf, sufentanil; TPM, topiramate; URTI, upper respiratory tract infection; VPA, valproic acid*.

* *Latent period: the days between the febrile illness and onset of seizures*.

All patients experienced a non-specific febrile illness prior to SE. Fever preceded the first seizures with a median duration of 4 days, ranging from 2 to 5 days. Shortly after onset of seizures, within 24 h, the seizures rapidly progressed into SE or became frequent with loss of consciousness between the attacks. An obvious feature from the first EEG segment (2–15 h) was the heavy burden of seizure and most of them were either focal or subclinical with altered consciousness. Out of the 7 patients, 5 had seizures comprising >50% in a 1-h period. Notable EEG parameters are described below and summarized in Table 2.

**Table 2:** EEG features.

			**Patient 1**	**Patient 2**	**Patient 3**	**Patient 4**	**Patient 5**	**Patient 6**	**Patient 7**
Ictal	Seizure duration/Average seizure burden	1#	2–40 min/1–2/h	6–33 min/3/h	2–4 min/6/h	0.5–1.5 min/2/h	2–5 min/13/h	1 min/1/h	2–4 min/0–1/h
		2#	1–6min/20/h	Continuously (unable to count)	0.5–5.5 min/8/h	0.5–2 min/3–4/h	2–3 min/40/h	12 min/0–1/h	1.5–3 min/2–3/h
		3#	1–7.5 min/17/h	Continuously (unable to count)	/	/	2–3 min/15/h	3–17 min/2/h	1.5–3.5 min/5–6/h
		4#	No seizure	Several mins/5/h	No seizure	2 min/0–1/h	1 min/10/h	No seizure	No seizure
		5#	No seizure	No seizure	0.5–2 min/2/h	No seizure	2–3 min/5/h	No seizure	No seizure
	Seizure origin	1#	L/R/BO, L/RF, L/RP, RC	RO, RT	L/RF	LO, LT	L/RO	Generalized	L/RT
		2#	L/R/BO, L/RT, L Rolandic	LT, R Rolandic	RO	LO, LT	L/R/BF, L/R/BT, L/R/BO	RT, generalized	L/RT, L/RF
		3#	L/R/BO, L/R Rolandic	L/RP, L/RO, RC	/	/	BF, BC, BT, BO	L/RF	RT, RF
		4#	No seizure	L/R Rolandic, L/RO	No seizure	LO	LF, RO, L/R Rolandic	No seizure	No seizure
		5#	No seizure	No seizure	L/RF	No seizure	RF	No seizure	No seizure
	Spreading/Generalizing/Shifting	1#	Yes/Yes/No	Yes/Yes/No	Yes/Yes/No	Yes/No/No	Yes/Yes/Yes	NA/NA/No	Yes/Yes/No
		2#	Yes/Yes/Yes	Yes/No/Yes	Yes/Yes/No	Yes/No/No	Yes/Yes/No	Yes/Yes/No	Yes/Yes/No
		3#	Yes/No/Yes	Yes/No/Yes	/	/	Yes/Yes/No	Yes/Yes/Yes	Yes/No/Yes
		4#	No seizure	No/No/Yes	No seizure	Yes/No/No	Yes/No/No	No seizure	No seizure
		5#	No seizure	No seizure	Yes/Yes/No	No seizure	Yes/No/No	No seizure	No seizure
Inter -ictal	Background	1#	Unidentified	Diffuse continuous δ activity with epileptiform discharges	Unidentified	Diffuse continuous δ activity with epileptiform discharges	Unidentified	Diffuse continuous δ and θ activity	Diffuse continuous θ activity
		2#	Unidentified	Diffuse continuous δ activity with epileptiform discharges	Diffuse continuous δ activity	Diffuse continuous δ activity with epileptiform discharges	Unidentified	Diffuse continuous δ and θ activity	Diffuse continuous δ activity
		3#	Diffuse continuous δ activity	Diffuse continuous δ activity with epileptiform discharges	/	/	Unidentified	Diffuse continuous δ activity	Diffuse continuous δ activity
		4#	Diffuse continuous δ with less θ activity	Diffuse continuous δ activity with epileptiform discharges	Diffuse continuous δ with less θ activity	Diffuse continuous δ activity with epileptiform discharges	Diffuse continuous δ activity	Diffuse continuous δ activity	Diffuse continuous δ activity
		5#	Diffuse continuous δ with θ activity	Diffuse continuous θ activity	Posterior basic rhythm	Diffuse continuous θ with less δ activity	Diffuse continuous δ activity	Diffuse continuous δ with θ activity	Posterior basic rhythm
	Sleep spindles	1#	Yes	Unidentified	Yes	No	Unidentified	Yes	Yes
		2#	Unidentified	Unidentified	No	No	Unidentified	Yes	Unidentified
		3#	No	Unidentified	/	/	Unidentified	Yes	Unidentified
		4#	Yes	Unidentified	No	Yes	Unidentified	Yes	Unidentified
		5#	Yes	Yes	Yes	Yes	Yes	Yes	Yes

*L, left; R, right; B, bilateral; T, temporal; C, central; P, parietal; O, occipital; F, frontal; NA, not applicable*.

**Figure 1:**
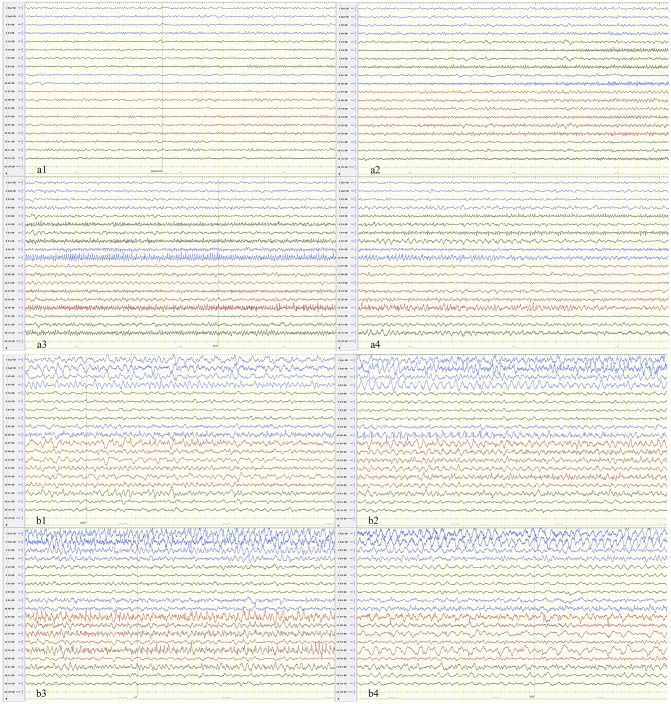
The seizure begins in the focal or multifocal regions with spread to the unilateral or bilateral hemisphere and shifting from one hemisphere to the contralateral. **(a)** Patient 1 and **(b)** patient 6.

We identified a specific ictal pattern of the seizures in 6 patients. This seizure pattern consisted of sharp (or spike) wave and/or sharp (or spike) slow wave complex of low to moderate amplitude, arising from unilateral or bilateral focal area, spreading to the same hemisphere and/or bilaterally with higher amplitude and faster/slower frequency, sometimes shifting from one hemisphere to the contralateral (Figure 1). Interictally, some patients displayed unidentified sleep architecture and diffuse delta-theta background slowing, with or without multifocal sporadic or periodic epileptiform discharges (sharp or spike waves, complex waves). These features might be undistinguishable from non-intermittent ictal activity in severe cases.

All patients experienced SE for 7–31 days (median 12; IQR 11; mean 16.6 ± 9.5) before KD initiation. In spite of the conventional management with a median of four AEDs and two continuous anesthetic agents before initiation of KD, the nearly continuous electrical or electroclinical seizures were poorly controlled or reappeared when anesthetic agents were tapered. Moreover, 2 patients received several anesthetics assistants (Fentanyl or its derivatives, Cisatracurium Besilate, Vecuronium) to control volcanic seizures. Three patients were under full mechanical ventilation due to severe respiratory depression. Similarly, other therapies such as intravenous immunoglobulin (IVIg) (in all patients) and Methylprednisolone (in 5 patients) were ineffective before initiation of KD. Before initiation of KD, most of the patients (except three) has stopped immunotherapy before or early after admission at our hospital. The three patients were continued on a low-dose steroid therapy even after KD initiated.

### Outcomes After KD Treatment

All patients achieved resolution of SRSE (Table 3) within a median of 5 days (IQR 3.5) after initiation of KD. The time to ketosis (defined as the level of serum β-hydroxybutyrate >1 mmol/L) of 6 of 7 patients ranged from 1 to 6 days, but 1 of 7 patients required 11 days to achieve ketosis after KD initiation. According to the EEG data, the seizure burden reduced, duration of seizure shortened, background recovered, and sleep architecture normalized gradually after KD initiation. Besides, 4 of 7 patients were weaned off general anesthetics within 15 days of KD initiation whereas one of them stopped anesthesia infusion before KD initiation to reduce respiratory secretions. The two patients who received intravenous KD were changed to enteral diet route gradually after resolution of SRSE.

**Table 3:** Patient in-hospital and long-term outcomes.

		**Patient 1**	**Patient 2**	**Patient 3**	**Patient 4**	**Patient 5**	**Patient 6**	**Patient 7**
**Clinical outcomes**								
Time to ketosis, *d*		3	2	11	1	5	1	3
Length of SE after KD initiation, *d*		6	6	1	0	10	5	4
Length of anesthesia after KD initiation, *d*		23	29	14	11	24	7	NA
No. of AEDs at hospital discharge		4	4	3	4	4	3	3
AEDs at hospital discharge		VPA, TPM, OXC, CLB	VPA, LEV, TPM, NZP	VPA, TPM, OXC	VPA, TPM, OXC, NZP	VPA, TPM, OXC, NZP	LEV, TPM, OXC	VPA, LEV, TPM
Adverse events		Hyperlipidemia, transient hyperamylasemia	Hyperlipidemia, diarrhea	Diarrhea	Hyperlipidemia	Hyperlipidemia	Diarrhea	Diarrhea
Total LOS, *d*		86	48	36	38	63	46	35
ICU LOS, *d*		61	32	19	23	40	33	20
**Follow-up**								
Duration of follow-up, *m*		13	20	31	40	6	14	11
Duration of KD, *m*		11	3	3	2	3	3	2
Seizure burden	At the end of KD	Monthly seizures (after controlled for 1 month)	No seizure	No seizure	Daily seizures (after controlled for 2 weeks)	Monthly seizures (after controlled for 2 months)	No seizure	No seizure
	At most recent follow-up	Monthly seizures	No seizure	Monthly seizures	Monthly seizures	Monthly seizures	Monthly seizures	No seizure
mRS score	At the end of KD	4	0	0	4	3	4	0
	At most recent follow-up	4	0	0	3	1	3	0

*mRS, modified Rankin Scale; NA, not applicable (because the anesthesia had been withdrawn before KD initiation in patient 7)*.

There was no severe acidosis (${\text{HCO}}_{3}^{-}$ <17 mmol/L) or hypoglycemia during KD therapy. Seven patients showed mild anomalies in their laboratory tests (Table 3). Of the 7 patients, 3 developed hyperlipemia or experienced diarrhea while 1 of 7 patients developed both conditions. Serum amylase increased to 389.7 mmol/L in one patient who was given IV KD, but normalized after adjustment of the proportion of KD ingredients, carbohydrate and by changing to enteral route. On discharge, all patients were followed up at the pediatric neurology outpatient clinics (Table 3). The number of AEDs used at hospital discharge ranged from 3 to 4 drugs. Duration from the time of discharge to the most recent follow-up ranged from 6 to 40 months. No patient remained on KD at the latest follow-up. Of 7 patients, one patient continued the diet for nearly 1 year compared to 6 patients who discontinued KD in 3 months. Among them, one patient had a recurrence of intractable epilepsy after about 2 weeks of treatment. The patient was, therefore, put on therapy of vagus nerve stimulation (VNS). The remaining patients were free of seizure attacks during the acute phase (4 were seizure-free, and one had intermittent seizures). At the time when KD was stopped, 4 of 7 patients had a mRS score ≤ 3, and the rate increased to 6 of 7 at the latest follow-up. A shorter duration of SE before KD initiation reflected a lower mRS score in the subsequent period (*p* = 0.012, *r* = 0.866). The recovery of phycomotor function was diverse among the patients, 3 cases (patient 2,3,7) were almost cured, 1 case (patient 5) had a significant improvement, and 3 cases (patient 1,4,6) showed a moderate improvement.

## Discussion

Although the clinical manifestations and disease course of FIRES have been well-defined in the literatures, this sudden and severe epileptic encephalopathy is still challenging. Patient with FIRES are often diagnosed using the exclusion diagnosis method, and this explains why patients with FIRES are often diagnosed at a late stage due to lack of early disease markers. FIRES is characterized by repeated and prolonged seizures, and patients with FIRES are always in prolonged pharmacologic coma which is often accompanied with medical complications. It is important to develop available diagnostic tools to facilitate prompt diagnosis and design effective therapies to improve the management and prognosis of this condition.

This study found that EEG monitoring is an effective method of diagnosing FIRES based on recordings of explosive seizures (most were subclinical) with high frequency. At the peak hours of SE, we identified a characteristic EEG pattern consisting of focal ictal activity of small to moderate amplitude at the onset of the seizure, which evolved into higher amplitude and faster/slower frequency, spreading unilaterally and/or bilaterally, sometimes shifting from one hemisphere to the contralateral. These features can be used for early recognition and intervention of FIRES.

Currently, there are no effective treatments for FIRES. Several case studies have shown that AEDs and immunological therapy do not satisfactorily control FIRES. And other studies have raised that the prolonged burst-suppression coma may be associated with a more severe course (2). Interestingly, some children with FIRES displayed good response to KD. Nine studies including 26 patients have reported this phenomenon (7, 10, 13, 17–22). The effective rate (>50% seizure reduction) was 62%. In this study of 7 children with FIRES and SRSE, we found that immunotherapy and AEDs could not control this condition, but KD treatment produced significant therapeutic efficacy and safety, which is consistent with previous reports.

It is worth noting that the clinical presentation of patients in this study shows that early initiation of KD improves outcomes of FIRES. In all patients, the frequency of seizures at the last follow-up was similar, and seizures were reduced by ≥75% in all patients except one who converted to VNS. However, their functional outcomes were dissimilar. A shorter duration of SE in the acute phase of patients indicated a lower mRS score in the subsequent period (*p* = 0.012, *r* = 0.866). Patients (2, 3, 5, 7) who had an SE duration of < 15 days before initiation of KD had a better prognosis with mRS scores ≤ 3 at the end of KD and ≤ 1 at the last follow up. In these patients, it is important to recognize FIRES symptoms early using specific EEG pattern and initiate KD treatment. KD therapy resolved SRSE successfully unlike other treatments (immunotherapy, four or more AEDs, and at least one general anesthetic agent). This indicates that KD can prevent damage from excitotoxicity processes, inhibit secondary processes induced by initial excitotoxicity, prevent multiple complications due to prolonged anesthesia and unconsciousness, increase alertness of patients and decrease the need for invasive respiratory support.

Generally, KD can be administered through enteral route (by mouth or tubes). But patients with conditions such as FIRES often suffer from gastrointestinal problems caused by prolonged use of AEDs, general anesthetics and mechanical ventilation. Some of the gastrointestinal problems include vomiting, severe diarrhea, and intestinal bleeding. Therefore, administration of drugs using enteral route may worsen these complications. In fact, in the presence of these conditions, enteral intake of KD is compromised and ketosis is impaired, making KD diet unsafe and inefficient (23). In this study, KD was given as parenteral nutrition to 2 children with FIRES and SRSE (patient 1 and 5) who required bowel rest due to upper gastrointestinal hemorrhage or gastroplegia. Thereafter, they were converted to enteral diet successfully. This study has demonstrated that KD can be effectively applied to patients with intestinal failure who need to start KD early.

The major limitations of this study should be discussed. This is a retrospective study containing a small number of patients. Considering the severity of patients with SRSE, we were unable to include a group of patients with SRSE and FIRES who were not put on KD treatment during the same period for comparison. Moreover, a combination of treatments comprising AEDs, immunotherapy and KD was used, making it difficult to isolate the exact benefit of a single intervention while excluding other treatments. Nevertheless, considering the rapid response (resolution of SE in clinical course and improvement of EEG features on assistant examination) in acute phase and good prognosis of patients in this study, we conclude that KD treatment is effective for FIRES patients. More studies are needed to reveal the exact mechanism of KD in FIRES, define the optimal timing and propose a protocol of KD application.

## Conclusions

In summary, this study shows that KD has a therapeutic effect on FIRES, especially in the acute phase as it can resolve SRSE. Early identification of FIRES and prompt initiation of KD therapy may improve the prognosis of this condition. Unique EEG features of FIRES may aid early diagnosis. Intravenous KD is a reasonable alternative for patients who cannot take KD enterally due to intestinal failure.

## Author Contributions

PP, LM, and JP conceived the study. XD and FH provided the clinical information and PP checked. CC, XW, and JP analyzed the EEG data. PP drafted the initial manuscript which was edited by LM, SG, FY, and JP.

### Conflict of Interest Statement

The authors declare that the research was conducted in the absence of any commercial or financial relationships that could be construed as a potential conflict of interest.
